# Prognostic value of the TCGA molecular classification in uterine carcinosarcoma

**DOI:** 10.1002/ijgo.13937

**Published:** 2021-10-11

**Authors:** Antonio Travaglino, Antonio Raffone, Diego Raimondo, Damiano Arciuolo, Giuseppe Angelico, Michele Valente, Giulia Scaglione, Nicoletta D’alessandris, Paolo Casadio, Frediano Inzani, Antonio Mollo, Angela Santoro, Renato Seracchioli, Gian Franco Zannoni

**Affiliations:** ^1^ Gynecopathology and Breast Pathology Unit Department of Woman’s Health Science Agostino Gemelli University Polyclinic Rome Italy; ^2^ Pathology Unit Department of Advanced Biomedical Sciences School of Medicine University of Naples Federico II Naples Italy; ^3^ Gynecology and Obstetrics Unit Department of Neuroscience, Reproductive Sciences and Dentistry School of Medicine University of Naples Federico II Naples Italy; ^4^ Division of Gynecology and Human Reproduction Physiopathology Department of Medical and Surgical Sciences (DIMEC) IRCCS Azienda Ospedaliero‐Univeristaria di Bologna S Orsola Hospital University of Bologna Bologna Italy; ^5^ Gynecology and Obstetrics Unit Department of Medicine, Surgery and Dentistry "Schola Medica Salernitana" University of Salerno Baronissi Italy; ^6^ Catholic University of Sacred Heart Rome Italy

**Keywords:** cancer, endoscopic surgery, gyne‐oncology, laparoscopy, mortality

## Abstract

**Background:**

The TCGA molecular groups of endometrial carcinoma are “POLE‐mutated” (POLEmut), “microsatellite‐instable/mismatch repair‐deficient” (MSI/MMRd), “TP53‐mutated/p53‐abnormal” (TP53mut/p53abn), and “no specific molecular profile” (NSMP).

**Objective:**

Prognostic assessment of the TCGA groups in uterine carcinosarcoma (UCS).

**Search strategy:**

Systematic review from January 2000 to January 2021.

**Selection criteria:**

Studies assessing the TCGA groups in UCS.

**Data collection and analysis:**

Progression‐free survival (PFS) and overall survival (OS) were assessed by Kaplan–Meier and Cox analyses (reference: TP53mut/p53abn group) and compared with endometrioid and serous carcinomas (original TCGA cohort), with a significant *P* < 0.050.

**Main results:**

Five studies with 263 UCS were included. Compared with TP53mut/p53abn UCS, MSI/MMRd UCS showed significantly better PFS (*P* < 0.001) but similar OS (*P* = 0.788), whereas NSMP UCS showed similar PFS (*P* = 0.936) and OS (*P* = 0.240). Compared with their endometrioid/serous counterparts, NSMP and TP53mut/p53abn UCS showed significantly worse PFS (*P* < 0.001 and *P* = 0.004) and OS (*P* < 0.001 and *P* < 0.001), while MSI/MMRd UCS showed similar PFS (*P* = 0.595) but significantly worse OS (*P* < 0.001). The POLEmut group showed neither recurrences nor deaths in both the UCS and the endometrioid/serous carcinoma cohorts.

**Conclusion:**

POLEmut UCS show excellent prognosis, whereas TP53mut/p53abn and NSMP UCS show a prognosis even worse than that of TP53mut/p53abn endometrioid/serous carcinomas. The prognosis of MSI/MMRd UCS remains to be defined.

## Abstract 2

POLEmut UCS show the same excellent prognosis as POLEmut endometrioid carcinomas, while TP53mut/p53abn and NSMP UCS show a prognosis even worse than TP53mut/p53abn endometrioid/serous carcinomas.

## 1 INTRODUCTION

Uterine carcinosarcoma (UCS) is a biphasic neoplasm constituted by an epithelial component and a stromal component, both of which are malignant and typically high‐grade. The epithelial component reflects the endometrial carcinoma histotypes, such as serous (most common), endometrioid, clear cells, mixed, and undifferentiated. The stromal component may be “non‐otherwise specified” or reflect the typical uterine sarcomas; in these cases, the stromal component is defined as “homologous”. If a differentiation towards extrauterine mesenchymal tissues is present, the stromal component is defined as “heterologous”.^1^, ^2^

The classification of UCS has changed over time. Indeed, UCS had previously been listed among the “mixed Müllerian tumors” and was lumped together with uterine sarcomas in terms of staging and treatment.^3^, ^4^ In recent decades, it has emerged that UCS represents a carcinoma with secondary sarcomatous transformation.^2^ Therefore, UCS is now classified as a subtype of endometrial carcinoma and is staged and managed accordingly.^1^, ^5^, ^6^ The rise of The Cancer Genome Atlas (TCGA) molecular classification has brought about a revolution in the prognostic stratification of endometrial carcinoma. The TCGA study and subsequent studies have identified four molecular prognostic groups: the “POLE‐mutated” (POLEmut) group, characterized by excellent prognosis; the “microsatellite‐instable/mismatch repair‐deficient” (MSI/MMRd) group and the “no specific molecular profile” (NSMP) group, characterized by intermediate prognosis; and the “TP53‐mutant/p53‐abnormal” (TP53mut/p53abn) group, characterized by poor prognosis.^7^, ^8^, ^9^, ^10^, ^11^, ^12^ However, the original 2013 TCGA study only included endometrioid and serous carcinomas,^7^ and the prevalence and prognostic value of the TCGA groups have been shown to vary across the different histotypes.^13^, ^14^, ^15^, ^16^, ^17^

Among UCS, the vast majority (>70%) fall into the TP53mut/p53abn group, which is consistent with its aggressive behavior.^16^ However, the prognostic value of the other TCGA groups in UCS is not well defined. Moreover, it is unclear if TP53mut/p53abn UCS have a prognosis similar to their endometrioid and serous counterparts. The aim of this quantitative systematic review was to assess the prognostic value of the TCGA groups in UCS and to compare the prognosis of the TCGA groups between UCS and the original 2013 TCGA cohort.

## 2 MATERIALS AND METHODS

Methods of this study were defined *a priori*, based on previous studies.^16^, ^18^ Each review stage was carried out by two independent authors; disagreements, if any, were resolved by consensus. The PRISMA statement^19^ was followed to report this study.

### 2.1 Search strategy and study selection

Four electronic databases (Scopus, ISI Web of Science, PubMed, Google Scholar) were searched from January 2000 to January 2021 for all studies assessing the TCGA molecular signatures in UCS. We adopted the following word combination: (uterine OR endometrial) AND (carcinosarcoma OR mixed malignant Müllerian) AND (TCGA OR ProMisE OR ultramutated OR POLE OR hypermutated OR mismatch OR microsatellite OR copy number OR TP53 OR p53). We also assessed the reference lists of eligible studies. We included all studies that assessed the TCGA groups and survival outcomes in UCS. Given the rarity of POLEmut UCS,^16^ we also included studies that did not assess POLE status. Exclusion criteria, defined a priori, were: sample size less than 10, data not extractable, reviews.

### 2.2 Data extraction

PICO^18^ of our study were: P (population) = women with UCS; I (intervention or risk factor) = POLEmut, MSI/MMRd and NSMP groups; C (comparator) = TP53mut/p53abn group; O (outcome) = progression‐free survival (PFS) and overall survival (OS). The TP53mut/p53abn group was used as reference (comparator) because it is the most represented group in UCS. Data were extracted without modifications.

### 2.3 Risk of bias assessment

The QUADAS‐2^20^ were used to assess the risk of bias within studies, as previously described.^16^, ^18^ Four domains were assessed: (1) “patient selection” domain (were selection criteria and period of enrollment reported and adequate?); (2) “index test” domain (were immunohistochemical and/or molecular methods reported and adequate); (3) “reference standard” domain (were follow‐up data reported and adequate?); and (4) “flow” (were all patients assessed for the TCGA classification and survival outcomes?). The risk of bias was categorized as “low”, “unclear”, or “high” as previously described.^16^, ^18^

### 2.4 Data analysis

PFS and OS for each TCGA group in UCS were represented graphically by using Kaplan–Meier curves. Cox regression survival analysis was used to calculate hazard ratio (HR) for recurrence and death in the TCGA group of UCS. Two analyses were performed: in the first analysis, HR was calculated in each TCGA group of UCA by using the TP53mut/p53abn group as reference; in the second analysis, each TCGA group of UCS was compared with the TCGA groups in the original TCGA series published in 2013, which included 232 patients with endometrial carcinoma (endometrioid, serous, and mixed). A *P* value less than 0.05 was considered significant. Statistical Package for Social Science (SPSS) 18.0 package (IBM Corp., Armonk, NY, USA) was used for the analyses.

## 3 RESULTS

### 3.1 Study selection and characteristics

Five studies^21^, ^22^, ^23^, ^24^, ^25^ with 263 UCS patients were included (Figure S1). The POLEmut group was assessed in three studies by using *POLE* sequencing. The MSI/MMRd group was assessed by MMR immunohistochemistry in three studies and by MSI testing in two studies. The TP53mut/p53abn group was assessed by p53 immunohistochemistry in two studies, by *TP53* sequencing in two studies and by both methods in the remaining study. Characteristics of the included studies are reported in Table 1.

**TABLE 1:** Characteristics of the included studies

Study	Country	Period of enrollment	Sample size	POLEmut		MSI/MMRd		TP53mut/p53abn		NSMP
				Test	Positive (%)	Test	Positive (%)	test	positive (%)	positive (%)
McConechy 2015	Canada	Unclear	30	Mol	1 (3.3)	IHC	1 (3.3)	mol, IHC	23 (76.7)	5 (16.7)
Cherniak 2017	USA	Unclear	57	Mol	1 (1.8)	Mol	2 (3.5)	mol	50 (87.7)	4 (7)
Jones 2019	USA	2007–2012	27	Not assessed	Not assessed	IHC	12 (44.4)	IHC	11 (40.7)	4 (14.8)
Gotoh 2019	Japan	1998–2015	92	Mol	10 (10.9)	Mol	24 (26.1)	mol	49 (53.3)	9 (9.8)
Saijo 2019	Japan	2007–2017	57	Not assessed	Not assessed	IHC	6 (10.5)	IHC	34 (59.6)	17 (29.8)

Abbreviations: IHC, immunohistochemistry; Mol, molecular analysis.

### 3.2 Risk of bias assessment

For the patient selection domain, three studies were considered at low risk of bias and two studies at unclear risk (period of enrollment not reported). For the index test domain, three studies were considered at low risk of bias and two studies at unclear risk (*POLE* mutations not assessed). For the “reference standard” domain, all studies were considered at low risk of bias. For the “flow” domain, the risk of bias was considered low for four studies and unclear for one study (only part of the cases was assessed by MMR immunohistochemistry). Authors’ judgments are reported in Figure S2.

### 3.3 Prognostic analysis

**FIGURE 1:**
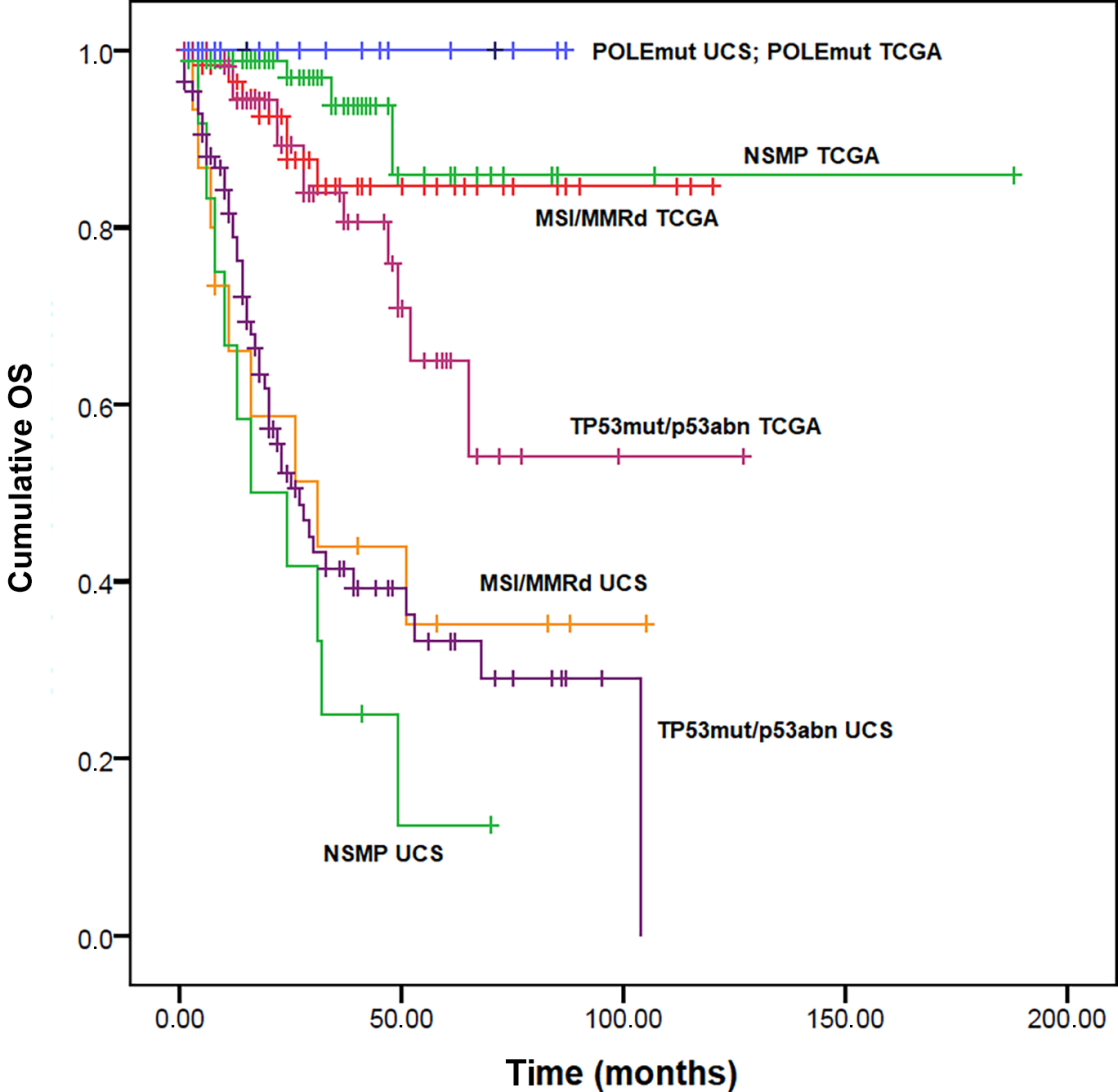
Kaplan–Meier curves for progression‐free survival in uterine carcinosarcoma and in the 2013 TCGA cohort, stratified based on the four TCGA molecular prognostic groups [Colour figure can be viewed at wileyonlinelibrary.com]

In the PFS analysis, none of the POLEmut carcinomas progressed among the UCS and in the 2013 TCGA cohort. In UCS, MSI/MMRd cases showed significantly better PFS than TP53mut/p53abn cases, with an HR of 0.19 (95% confidence interval [CI] 0.08–0.46; *P* < 0.001), but no significant difference was found between NSMP and TP53mut/p53abn cases (HR 1.02, 95% CI 0.59–1.78; *P* = 0.936). Compared with the correspondent groups in the 2013 TCGA cohorts, MSI/MMRd UCS showed similar PFS (HR 0.75, 95% CI 0.26–2.19; *P* = 0.595), while NSMP and TP53mut/p53abn UCS showed significantly worse PFS, with HR of 5.31 (95% CI 2.44–11.59; *P* < 0.001) and 2.18 (95% CI 1.29–3.69; *P* = 0.004), respectively (Figure 1).

**FIGURE 2:**
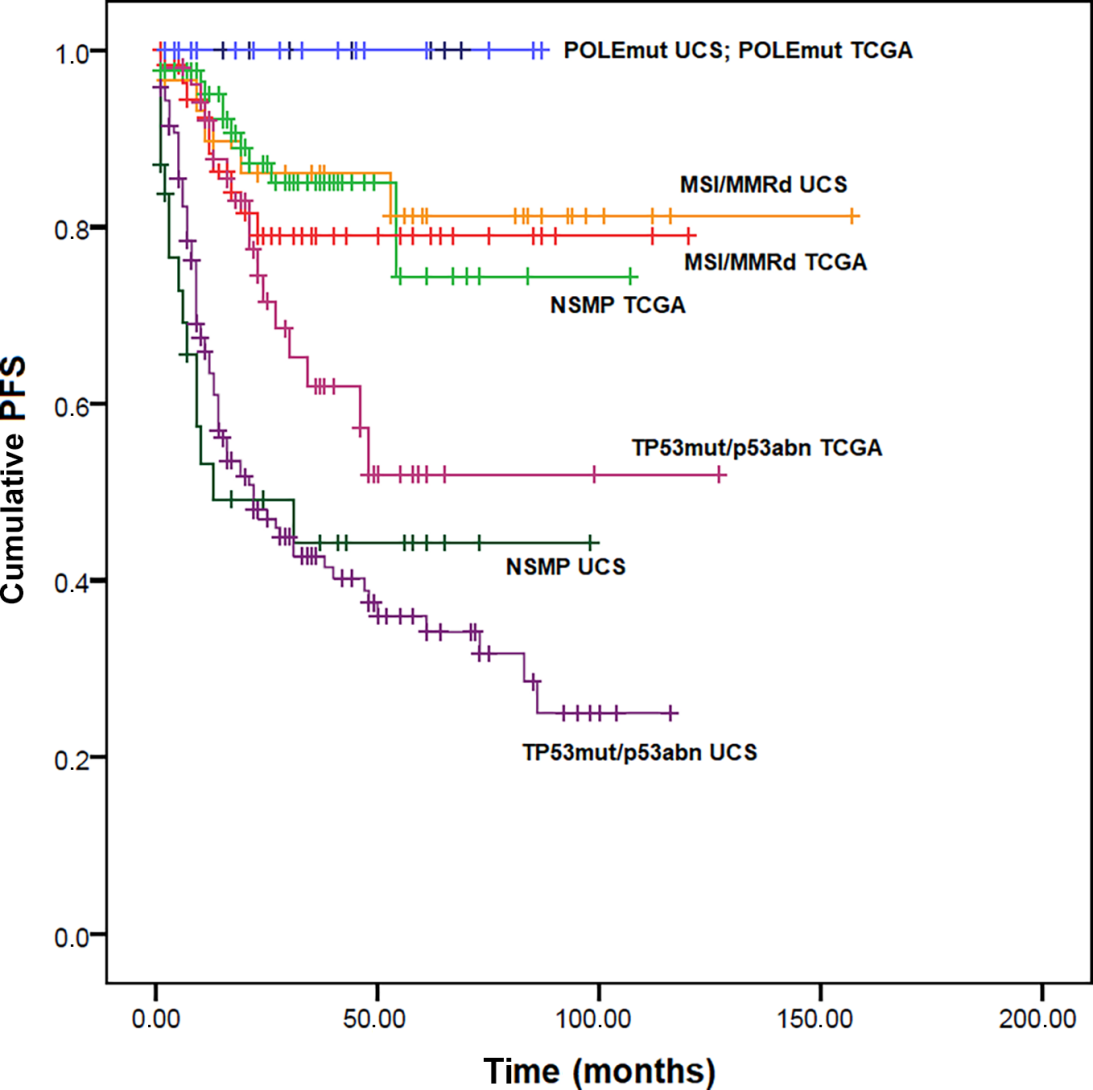
Kaplan–Meier curves for overall survival in uterine carcinosarcoma and in the 2013 TCGA cohort, stratified based on the four TCGA molecular prognostic groups [Colour figure can be viewed at wileyonlinelibrary.com]

In the OS analysis, none of the POLEmut patients died among the UCS and in the 2013 TCGA cohort. In UCS, MSI/MMRd and NSMP cases showed similar OS to the TP53mut/p53abn cases, with HR of 0.91 (95% CI 0.44–1.87; *P* = 0.788) and 1.51 (95% CI 0.76–2.99; *P* = 0.240), respectively. Compared with the correspondent groups in the 2013 TCGA cohorts, MSI/MMRd, NSMP, and TP53mut/p53abn UCS showed significantly worse OS, with HR of 5.90 (95% CI 2.19–15.86; *P* < 0.001), 22.02 (95% CI 6.90–70.27; *P* < 0.001), and 3.51 (95% CI 1.86–6.64; *P* < 0.001), respectively (Figure 2). Survival analysis results are reported in Tables 2 and 3.

**TABLE 2:** Progression‐free survival and overall survival in the TCGA groups of uterine carcinosarcoma, using the p53abn group as reference

TCGA group (UCS)	Progression‐free survival			Overall survival		
	HR	95% CI	*P* value	HR	95% CI	*P* value
POLEmut	NV	NV	NV	NV	NV	NV
MMRd	0.19	0.08–0.46	<0.001	0.91	0.44–1.87	0.788
NSMP	1.02	0.59–1.78	0.936	1.51	0.76–2.99	0.240
p53abn (reference)	1.00	—	—	1.00	—	—

Abbreviations: CI, confidence interval; HR, hazard ratio; UCS, uterine carcinosarcoma.

^a^ NV, not evaluable, because no event occurred in POLEmut cases.

**TABLE 3:** Progression‐free survival and overall survival in the TCGA groups of uterine carcinosarcoma, using the correspondent groups in the 2013 TCGA cohort as reference

TCGA group (UCS vs 2013 TCGA cohort)	Progression‐free survival			Overall survival		
	HR	95% CI	*P* value	HR	95% CI	*P* value
POLEmut	NV^a^	NV	NV	NV	NV	NV
MMRd	0.75	0.26–2.19	0.595	5.90	2.19–15.86	<0.001
NSMP	5.31	2.44–11.59	<0.001	22.02	6.90–70.27	<0.001
p53abn	2.18	1.29–3.69	0.004	3.51	1.86–6.64	<0.001

Abbreviations: CI, confidence interval; HR, hazard ratio; UCS, uterine carcinosarcoma.

^a^ NV, not evaluable, because no event occurred in POLEmut cases.

## 4 DISCUSSION

This study showed that POLEmut UCS had the same excellent prognosis as POLEmut endometrioid carcinomas, whereas TP53mut/p53abn and NSMP UCS had a prognosis even worse than TP53mut/p53abn endometrioid/serous carcinomas. MSI/MMRd UCS showed similar PFS but worse OS than their endometrioid counterpart.

In the last decades, the risk stratification of endometrial carcinoma has been based on poorly reproducible histologic features and on simplistic clinical classifications.^26^, ^27^, ^28^, ^29^ Since their publication in 2013, the TCGA findings have progressively changed the approach to the study of endometrial carcinoma.^6^, ^7^, ^30^ The “Proactive Molecular Risk Classifier for Endometrial Cancer” (ProMisE) is a surrogate of the TCGA classification, which has allowed a wider applicability of the TCGA groups.^8^, ^9^, ^10^ The ProMisE has indeed introduced the use of mismatch repair protein immunohistochemistry as a surrogate of microsatellite instability molecular testing, and of p53 immunohistochemistry as a surrogate of somatic copy number alteration testing.^7^, ^8^, ^9^, ^31^, ^32^ In 2020, the TCGA prognostic groups have been integrated in the European Society of Gynaecological Oncology/ European Society for Therapeutic Radiotherapy and Oncology/ European Society of Pathology (ESGO/ESTRO/ESP) guidelines for management of endometrial carcinoma. According to such guidelines, all POLEmut carcinomas up to FIGO (the International Federation of Gynecology & Obstetrics) Stage II should be considered at low risk, while all TP53mut/p53abn carcinomas with myometrial invasion should be considered at high risk. In contrast, the risk stratification of the MSI/MMRd group and NSMP group is affected by pathologic prognostic factors such as FIGO grade, histotype, lymphovascular space invasion, and deep myometrial invasion.^6^ Although such integrated classification represents an important step forward in the management of endometrial carcinomas, there are still some points that should be clarified. Despite being lumped together in the guidelines, the NSMP and MSI/MMRd groups may diverge in selected subsets of endometrial carcinomas. In particular, the NSMP group seems to be much more prognostically heterogeneous than the MSI/MMRd group.^7^, ^8^, ^9^, ^10^, ^11^, ^12^, ^21^, ^22^, ^33^, ^34^, ^35^ Furthermore, the ESGO/ESTRO/ESP guidelines place all non‐endometrioid carcinomas in the same risk group regardless of the TCGA signature (except for the POLEmut group).^6^ Possible differences between different histotypes within the TP53mut/p53abn group are also disregarded.

In this study, we assessed the prognosis of the TCGA groups in UCS compared with their counterparts in the original 2013 TCGA cohort, which comprised endometrioid and serous carcinomas.^7^ We found that POLEmut UCS showed neither recurrences nor deaths, a result that was superimposable on the POLEmut endometrioid carcinomas of the 2013 TCGA cohort. The excellent prognosis of the POLEmut groups was also demonstrated in clear‐cell carcinomas, mixed carcinomas, and even undifferentiated/dedifferentiated carcinomas.^34^, ^35^, ^36^, ^37^ These findings agree with the ESGO/ESTRO/ESP guidance, supporting that all POLEmut endometrial carcinomas should be considered at low risk regardless of histotype.^6^

Regarding the TP53mut/p53abn group, we found that UCS of this group showed poor PFS and poor OS, consistently with what observed in other histotypes.^7^, ^33^, ^34^, ^35^, ^37^, ^38^, ^39^ In addition, we found that TP53mut/p53abn UCS showed significantly poorer PFS and OS than their 2013 TCGA counterparts (mostly serous carcinomas). Given that the TP53mut/p53abn group is the most represented in UCS, this finding supports that UCS is overall more aggressive than the prototypical type II endometrial cancer. This is in agreement with previous studies that suggested a worse prognosis for UCS compared to serous and clear cell carcinoma.^40^, ^41^, ^42^ Remarkably, the NCCN guidelines recommend adjuvant therapy for UCS even in the case of disease limited to the endometrium with no residual on the final hysterectomy specimens; such recommendation is not made for serous and clear cell carcinoma.^5^

Regarding the NSMP group, we found that UCS of this group showed a poor prognosis, similar to that of TP53mut/p53abn UCS and significantly poorer than that of TP53mut/p53abn endometrioid/serous carcinomas. This confirms the extreme variability in the prognosis of the NSMP group, which appears good‐to‐intermediate in low‐grade endometrioid carcinomas, intermediate in high‐grade endometrioid carcinomas, and poor in non‐endometrioid carcinomas.^7^, ^33^, ^37^, ^39^ This also supports a worse prognosis for UCS compared with the classical type II endometrial carcinomas, as discussed above.

The prognostic analysis of the MSI/MMRd group showed discordance between PFS and OS in UCS. In the PFS analysis, MSI/MMRd UCS showed an intermediate prognosis similar to that of endometrial carcinomas (all endometrioid) of the same group in the 2013 TCGA cohort. In the OS analysis, MSI/MMRd UCS showed a very poor prognosis similar to that of TP53mut/p53abn and NSMP UCS and worse than any 2013 TCGA group. By assessing the possible causes for such discrepancy, we noted that most MSI/MMRd UCS in the OS analysis derived from a study with an unusually high prevalence of the MSI/MMRd signature.^23^ Indeed, almost half (44.4%) of UCS in that study were MSI/MMRd, against an average percentage less than 10%.^16^ Such a high percentage of MSI/MMRd cases is analogous to that observed in undifferentiated/dedifferentiated carcinoma.^14^ Interestingly, dedifferentiated carcinoma may show morphologic and immunohistochemical overlap with UCS.^43^ Indeed, both are biphasic tumors with an overt carcinomatous component and a dyshesive malignant component.^1^, ^14^, ^16^ The dyshesive component of dedifferentiated carcinoma may show cell spindling, rhabdoid morphology, and myxoid stroma, which mimic a sarcomatous component.^44^ Furthermore, UCS may sometimes show a low‐grade carcinoma component, as is typically observed in dedifferentiated carcinoma, whereas the latter may show a high‐grade carcinoma component, which is more typical of UCS.^25^, ^45^ In dedifferentiated carcinoma, the MSI/MMRd group seems to have a poor prognosis similar to that of the TP53mut/p53abn and NSMP groups^18^; by contrast, in other non‐endometrioid carcinomas, the MSI/MMRd signature is associated with improved prognosis.^33^, ^34^, ^35^, ^37^, ^46^ On this account, we hypothesize that the poor prognosis of MSI/MMRd UCS in our analysis might be a result of the inclusion of dedifferentiated carcinomas. Recently, it has been suggested that the highly aggressive behavior of undifferentiated/dedifferentiated carcinomas may be due to mutations in proteins of the SWI/SNF complex (ARID1B, SMARCA4/BRG1, SMARCB1/INI1), which occur in about two‐thirds of cases^18^, ^45^, ^47^; the loss of these proteins on immunohistochemistry seems to be a specific diagnostic marker of undifferentiated/dedifferentiated carcinoma.^48^ We believe that further studies are necessary to define the biologic behavior of MSI/MMRd. For this purpose, it would be advisable to perform SWI/SNF protein immunohistochemistry in all cases that may raise the possibility of a dedifferentiated carcinoma. Such a procedure would allow the exclusion of highly aggressive dedifferentiated carcinoma and achieve a more precise prognostic definition of MSI/MMRd UCS.

A limitation of our results is the low number of patients in the groups other than TP53mut/p53abn, especially in the POLEmut group and in the OS analysis. Furthermore, we had no sufficient data to perform a multivariate survival analysis, because not all studies reported individual clinical data. Finally, we could not review histologic and immunohistochemical findings to confirm the diagnosis of UCS.

In conclusion, the TCGA classification significantly stratifies OS and PFS in UCS. POLEmut UCS show an excellent prognosis similar to that of POLEmut endometrioid carcinomas, supporting their inclusion in the same low‐risk category for management purposes. TP53mut/p53abn and NSMP UCS show a very poor prognosis, seemingly even worse than that of serous carcinomas. Whether MSI/MMRd UCS should be considered intermediate‐risk like MSI/MMRd carcinomas remains to be defined; for this purpose, a screening with SWI/SNF protein immunohistochemistry would be useful to exclude highly aggressive dedifferentiated carcinomas, which may mimic UCS and confound the results.

## CONFLICTS OF INTEREST

The authors have no conflicts of interest.

## AUTHOR CONTRIBUTIONS

The study was conceived by AT, AR, DR, DA, AS, RS, and GFZ and the protocol was developed by AT, AR, DR, DA, GA, GS, AM, AS, RS, GFZ. Electronic search was oerfirned by GS and NDA; study selection by MV and GA; data extraction by AM and DR; risk of bias assessment by PC and FI; and data analysis by AT and AR. Disagreements were resolved by AT, AR, DR, DA, GA, MV, GS, NDA, PC, FI, AM, AS, RS, and GFZ. Interpretation was by AT, AR, DR, DA, PC, FI, AM, AS, RS, and GFZ. The first draft was written by DR, DA, GA, MV, GS, NDA, and FI and was revised by AT, AR, AS, RS, PC, AM, and GFZ. The study was supervised by AT, AR, AM, AS, RS, and GFZ.

## Supporting information

Fig S1

Fig S2

Supplementary Material
