# Supplementary figures and images for: Prognostic value of the TCGA molecular classification in uterine carcinosarcoma
Source: Int J Gynaecol Obstet. 2021 Oct 11;158(1):13–20. doi: 10.1002/ijgo.13937 (PMC9292561; doi:10.1002/ijgo.13937)

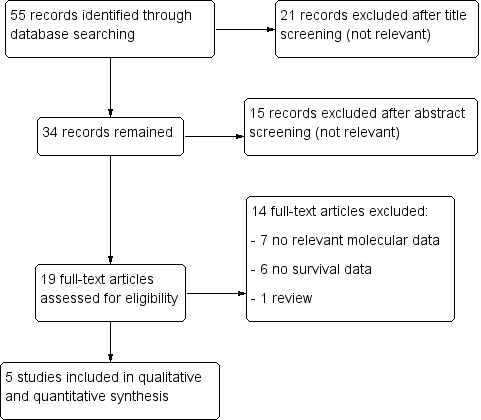

Supplement: Supplementary file 1 — Fig S1 [file IJGO-158-13-s001.png]

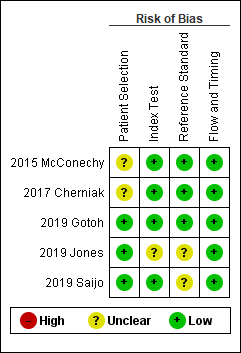

Supplement: Supplementary file 2 — Fig S2 [file IJGO-158-13-s002.png]
